# Energy dependence and angular dependence of an optically stimulated luminescence dosimeter in the mammography energy range

**DOI:** 10.1002/acm2.12041

**Published:** 2017-01-24

**Authors:** Ai Kawaguchi, Yuta Matsunaga, Shoichi Suzuki, Koichi Chida

**Affiliations:** ^1^ Department of Radiological Technology Graduate School of Medicine Tohoku University Sendai Japan; ^2^ Department of Radiology TOYOTA Memorial Hospital Toyota Japan; ^3^ Department of Imaging Nagoya Kyoritsu Hospital Nagoya Japan; ^4^ School of Health Sciences Fujita Health University Toyoake Japan

**Keywords:** angular dependence, energy dependence, mammography, optically stimulated luminescence dosimetry, point‐based dosimeter

## Abstract

This study aimed to investigate the energy dependence and the angular dependence of commercially available optically stimulated luminescence (OSL) point dosimeters in the mammography energy range. The energy dependence was evaluated to calculate calibration factors (CFs). The half‐value layer range was 0.31–0.60 mmAl (Mo/Mo 22–28 kV, Mo/Rh 28–32 kV, and W/Rh 30–34 kV at 2‐kV intervals). Mo/Rh 28 kV was the reference condition. Angular dependence was tested by rotating the X‐ray tube from −90° to 90° in 30° increments, and signal counts from angled nanoDots were normalized to the 0° signal counts. Angular dependence was compared with three tube voltage and target/filter combinations (Mo/Mo 26 kV, Mo/Rh 28 kV and W/Rh 32 kV). The CFs of energy dependence were 0.94–1.06. In Mo/Mo 26–28 kV and Mo/Rh 28–32 kV, the range of CF was 0.99–1.01, which was very similar. For angular dependence, the most deteriorated normalized values (Mo/Mo, 0.37; Mo/Rh, 0.43; and W/Rh, 0.58) were observed when the X‐ray tube was rotated at a 90° angle, compared to 0°. The most angular dependences of ± 30°, 60°, and 90° decreased by approximately 4%, 14%, and 63% respectively. The mean deteriorated measurement 30° intervals from 0° to ± 30° was 2%, from ± 30° to ± 60° was 8%, and from ± 60° to ± 90° was 40%. The range of energy dependence in typical mammography energy range was not as much as that in general radiography and computed tomography. For accurate measurement using nanoDot, the tilt needs to be under 30°.

## 1 Introduction

Mammography is the most effective method to screen for breast cancer. The breast includes skin, glandular tissue, adipose tissue, and areolar tissue. The glandular tissue is highly sensitive to some adverse effects of radiation.^1^ A prerequisite in patient dose management is that the benefits of screening should be considerably greater than the risks induced by the use of radiation.

Some point‐based dosimeters such as thermoluminescent dosimeters^2^ and radio photoluminescent glass dosimeters^3^ have been used to directly measure dosage in patient dose management. The energy range in mammography is very low, compared to that used in general radiography and computed tomography (CT). Therefore, these point‐based dosimeters usually require corrections for energy dependence.^4^, ^5^, ^6^

Optically stimulated luminescent (OSL) dosimeters were recently introduced as point‐based dosimeters into medical and environmental dosimetry. The OSL dosimeter was first adopted for medical dosimetry in radiation therapy.^7^, ^8^, ^9^, ^10^ Later, research widened to studying the feasibility of using OSL dosimeters in the diagnostic energy range.^11^, ^12^, ^13^, ^14^ The mechanism of optically stimulated luminescence and thermoluminescence are similar processes.^8^, ^15^ The structure of the dosimeter is composed of pure crystalline dielectric materials and contains a small quantity of dopants that form crystal‐lattice imperfections. These imperfections act as traps for electrons and holes. Electrons and holes are trapped by these energy traps after exposure to ionizing radiation. When the crystal is stimulated with a light‐emitting diode, for example, at a dosimeter readout, the electrons can be ejected out of traps and recombined with holes while emitting characteristic light proportional to the amount of the absorbed radiation dose.^8^

Currently, the only material used broadly in OSL dosimeters is aluminum oxide with carbon doping (Al_2_O_3_:C). One type of Al_2_O_3_‐based OSL dosimeter, the nanoDot (Landauer, Inc., Glenwood, IL, USA), is commercially available and is small, robust, and reusable. It has high sensitivity, and its density is near to that of a human body,^11^, ^12^, ^16^ making it a realistic choice for point measurements in diagnostic imaging.^13^, ^14^ Jursinic et al.^8^ reported that OSL dosimeters exhibit high precision and accuracy in measuring a dose, and they have no energy dependence, and no dependence on the irradiation angle in the radiation therapy energy range. Al‐Senan and Hatab^11^ investigated the feasibility of using commercially available OSL dosimeters in the diagnostic energy range including a part of the mammography energy range. The linearity test showed good linear response with *R* ^2^ > 0.99 and the angular dependence showed the maximum variation as a drop of approximately 70% at 90° at only 25 kV in the mammography energy range. The OSL dosimeters also had energy dependence and were recommended to acquire correction factors in the diagnostic energy range, however, the mammography energy range was not investigated. The energy dependence and angular dependence of the OSL dosimeter require further investigation to evaluate the feasibility of their use in the mammography energy range, based on the various tube voltage of each target and filter combinations.

In this study, we present an evaluation of two specific dosimetric characteristics in the mammography energy range of a commercial OSL dosimeter. We investigated energy dependence and angular dependence based on tube voltage of each target and filter combination.

## 2 Materials and methods

**Figure 1:**
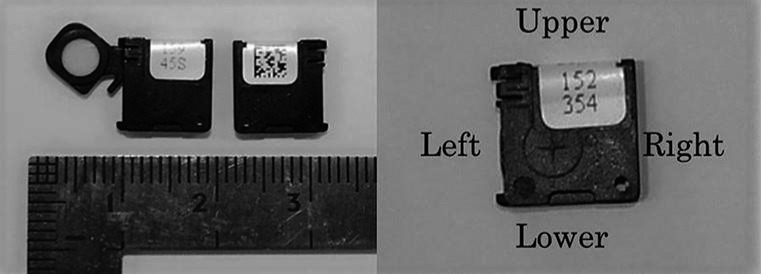
NanoDot dosimeter. The left panel shows two nanoDots, including the open front side of a nanoDot (left) revealing the aluminum oxide‐based (Al_2_O_3_:C) disk and the reverse side showing the closed dosimeter (right). The right panel shows the front of a nanoDot.

The OSL dosimeter system included InLight nanoDot OSL dosimeters (Landauer Inc., Glenwood, IL, USA) and the microStar reader (Landauer Inc.). The nanoDot consists of a 0.2‐mm‐thick aluminum oxide‐based (i.e., Al_2_O_3_:C) disk‐shaped detector with a diameter of approximately 5 mm encased in a light‐tight 10 × 10 × 2 mm^3^ plastic case that had a mass density of 1.03 g/cm^3^. Standard nanoDots were used in this study (± 10% variation in the labeled accuracy values provided by Landauer Inc.). Fig. 1 demonstrates the structure of the nanoDot device. The front of the nanoDot shows a printed number and the reverse shows the QR code.

NanoDots were read using the microStar Reader (Landauer Inc.), which has an array of light‐emitting diodes as the high‐intensity stimulation source. For all measurements, readings were performed 30 min after irradiation. Each nanoDot was read at least three times, and only the mean reading was utilized in the study. As part of the microStar Reader's quality control (QC) procedure, variations in the reader's sensitivity were checked daily by measuring the background signal (DRK), photomultiplier tube (PMT) counts from the^14^C source (CAL), and counts from PMT with the shutter open and the light‐emitting diodes on to indicate beam intensity (LED). The QC procedure was repeated three times, and the average counts were recorded to ensure that the variations were within the manufacturer's recommended limits: DRK < 30, and CAL and LED ± 10% of the reader's established average. The microStar Reader was calibrated using five pre‐irradiated nanoDots provided by the manufacturer, which had been exposed to known doses in the air, ranging from 0–1 Gy using an 80 kV beam with a half‐value layer (HVL) of 2.9 mmAl (which corresponds approximately to an effective energy of 33 keV) by conventional diagnostic radiology applications. The microStar Reader employs two calibration methods: low dose and high dose. The light‐emitting diode beam operates in the high‐power mode for low‐dose calibration, and in the low‐power mode for high‐dose calibration. The readouts in this research were performed in the low‐dose mode. All OSL counts reported indicate the PMT counts, as displayed by the reader. Following the reading, nanoDots were optically bleached by placing them under a 60 W fluorescent bulb for approximately 5 h. Optical bleaching cannot remove all signal counts and residual signal counts remain.^8^, ^17^ NanoDots were then read to ensure that they had been adequately bleached (i.e., to less than 100 counts). In all measurements, a reading was performed before irradiation and 0.5–2.0 h after irradiation. The signal counts of a measurement were reported as the difference between the postirradiation and preirradiation signal counts.

Irradiations were carried out using two mammography systems (Sepio: Shimadzu, Kyoto, Japan; and Amulet: Fujifilm, Tokyo, Japan). Sepio was used for measurements with a molybdenum target and molybdenum filter (Mo/Mo) combination, and a molybdenum target and rhodium filter (Mo/Rh) combination; in contrast, Amulet was used for measurements with only the tungsten target and rhodium filter (W/Rh) combination. A parallel‐plate ionization chamber (Model 9015, 1095–6M, 6 cm³; Radcal Corp., Monrovia, CA, USA) was used to estimate the irradiated dose. The ionization chamber was calibrated at a laboratory accredited by the Japan Quality Assurance Organization (Tokyo, Japan). To confirm the stabilization of the radiation output and the reproducibility of geometric arrangement for the ionization chamber, only the ionization chamber was irradiated under the same conditions of Mo/Rh 28 kV and 100 mAs on Sepio, and under W/Rh 32 kV and 250 mAs on Amulet, before every test.

### 2.A Energy dependence

**Figure 2:**
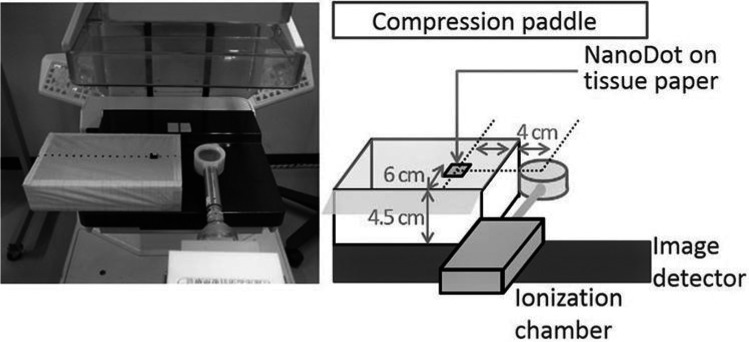
Experimental setup for energy dependence.

Three nanoDots were used to investigate energy dependence. Irradiations were performed under the following conditions: Mo/Mo 22–28 kV, Mo/Rh 28–32 kV, and W/Rh 30–34 kV at 2‐kV intervals. The mAs exposure conditions were modulated to approximately 10 mGy. Experimental setups for the nanoDot and the ionization chamber are shown in Fig. 2. A nanoDot and ionization chamber were placed symmetrically and laterally at 40 mm from the center, 45 mm above the breast support table, and 60 mm from the side of the chest wall on tissue paper. The compression paddle was located at the greatest distance possible from the image detector. The distance from the breast support table and side of the chest wall on tissue paper was based on the European Organization for Quality Assured Breast Screening and Diagnostic Services (EUREF) protocol.^18^ Measurements were repeated using three nanoDots and the ionization chamber. In this set of irradiations, Mo/Rh 28 kV was used as the reference condition and calibration factors (CFs) were calculated to evaluate energy dependence.

The CF for energy dependence was defined as^12^

(1) $$\text{CF}=\frac{S/D}{S_{ref}/D_{ref}}$$

where S is the nanoDot's signal count and D is the dose from the ionization chamber that was irradiated simultaneously. S_ref_ is the nanoDot's signal count under the reference conditions (i.e., Mo/Rh 28 kV) and D_ref_ is the dose from the ionization chamber that was irradiated simultaneously under the reference conditions.

An HVL measurement was performed with the addition of Al foil above the compression paddle, which was located at the longest possible distance from the image detector and intercepted the entire radiation field, and the ionization chamber was placed at the center point and 60 mm from the side of the chest wall on the breast support table as per the EUREF protocol.^18^ The ionization chamber was irradiated three times, and only the mean value was utilized. For every measurement, the HVL was calculated and the corresponding effective photon energy (keV) was estimated using data of the mass attenuation coefficient for Al.^19^

### 2.B Angular dependence

**Figure 3:**
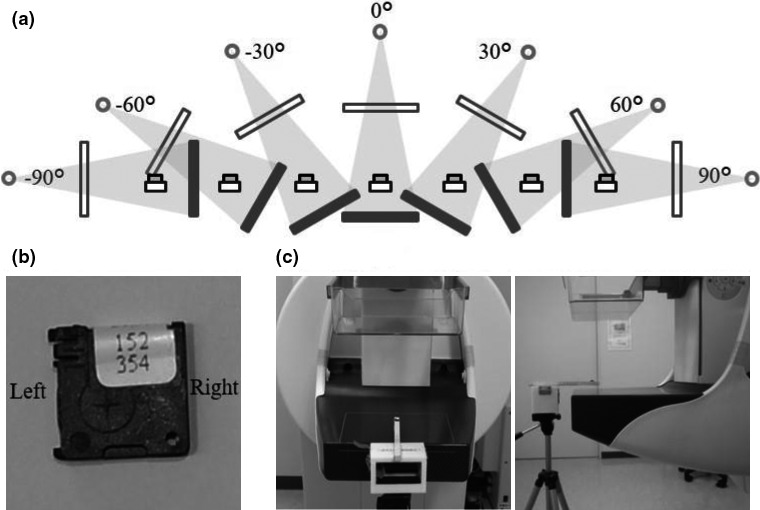
Experimental setup for angular dependence. (a) The upper experimental setup shows the measurement of a nanoDot at different X‐ray tube angles. (b) The measurement of the nanoDots and (c) the photo of the experimental setup.

The variability in the nanoDot response to incident X‐ray beams from various angles was investigated. Seven different angles were evaluated with eight nanoDots (one nanoDot was used as a control). Individual nanoDot sensitivity was corrected to acquire an average signal in counts/mGy. A nanoDot set on hard paper at the rotation isocenter of the X‐ray tube (isocenter points, 35 mm [Sepio] and 30 mm [Amulet] above the breast support table and 60 mm from the side of the chest wall according to the dosimetry method described in the EUREF protocol^18^, ^20^) such that the side with the printed serial number was facing upward [Fig. 3(b), (c)]. Irradiations were performed under the conditions of Mo/Mo 26 kV, Mo/Rh 28 kV and 100 mAs (Sepio) or W/Rh 32 kV and 250 mAs (Amulet). The X‐ray tube was rotated by −90°, −60°, −30°, 0°, 30°, 60°, and 90° [Fig. 3(a)]. The same cycle of irradiation, readout, and bleaching was repeated three times. Signal counts from angled nanoDots were normalized to the 0° signal counts, in which the detector's serial number was facing the beam.

## 3 Results

To confirm the reproducibility of the radiation output and geometric arrangement of the ionization chamber, the ionization chamber was irradiated under the conditions of Mo/Rh 28 kV and 100 mAs on Sepio and of W/Rh 32 kV and 250 mAs on Amulet before every test. The reproducibility as the coefficient of variation (CV) was 0.5% and 0.8% respectively.

### 3.A Energy dependence

**Figure 4:**
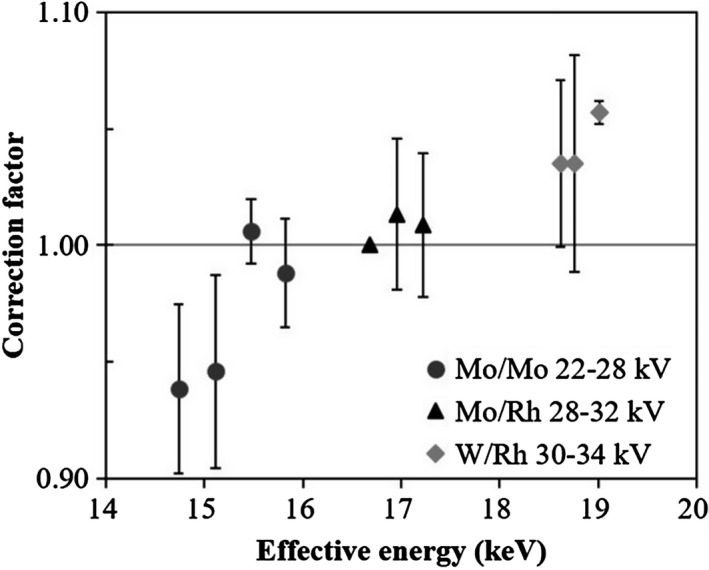
NanoDot correction factors as a function of effective photon energy. The error bars represent 95% confidence intervals from 3 nanoDots.

Fig. 4 demonstrates the obtained CFs for nanoDots at different measured effective energies from Mo/Mo 22–28 kV, Mo/Rh 28–32 kV, and W/Rh 30–34 for each 2‐kV increment, which corresponded to HVLs of 0.31–0.60 mmAl. The ranges of CFs were observed from 0.94 to 1.06. Table 1 lists the CFs, corresponding HVLs, calculated effective energy, and 95% confidence interval (CI).

**Table 1:** Estimated correction factors and standard deviations from three nanoDots with various target and filter combinations, tube voltages (kV), calculated half‐value layers, and corresponding effective energies (keV)

Target/filter	Tube voltage (kV)	HVL (mmAl)	Eeff (keV)	CF	95% CI
Mo/Mo	22	0.31	14.7	0.94	0.90–0.97
	24	0.33	15.1	0.95	0.90–0.99
	26	0.35	15.5	1.01	0.99–1.02
	28	0.37	15.8	0.99	0.96–1.01
Mo/Rh	28	0.42	16.7	1.00	1.00
	30	0.44	17.0	1.01	0.98–1.05
	32	0.45	17.2	1.01	0.98–1.04
W/Rh	30	0.56	18.6	1.03	1.00–1.07
	32	0.58	18.8	1.04	0.99–1.08
	34	0.60	19.0	1.06	1.05–1.06

CF, correction factor; HVL, half‐value layer; Eeff, effective energy; CI, confidence interval.

### 3.B Angular dependence

**Figure 5:**
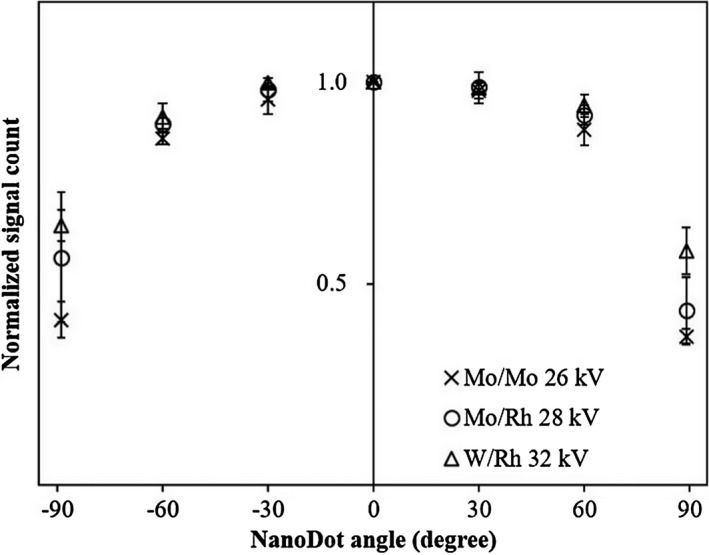
Results for angular dependence were compared between three tube voltage and target/filter combinations. Doses from angled nanoDots were normalized to the 0° signal count, in which the detector's serial number was facing the beam. The error bars represent the standard deviation from three measurements.

Fig. 5 shows the results for angular dependence with three tube voltage and target/filter combinations. The angular dependences of each angle of the Mo/Mo and 26 kV combination were the most deteriorated among three tube voltage and target/filter combinations. The nanoDot demonstrated the most deteriorated normalized values (Mo/Mo, 0.37; Mo/Rh, 0.43; and W/Rh, 0.58) when the X‐ray tube was rotated at a 90° angle, compared with 0° for all. The angular dependences at ± 60° were between 0.94 (minimum, W/Rh 60°) and 0.86 (maximum, Mo/Mo −60°). The angular dependences at ± 30° were between 0.997 (minimum, W/Rh −30°) and 0.96 (maximum, Mo/Mo −30°). The mean deteriorated measurement 30° intervals from 0° to ± 30° was 2%, from ± 30° to ± 60° was 8%, and from ± 60° to ± 90° was 40%.

## 4 Discussion

Commercially available OSL dosimeters were tested for the energy dependence and the angular dependence based on the tube voltage of each target and filter combination in the mammography energy range. Energy dependences were observed in approximately 10% of the typical mammography energy range. Angular dependence showed the most deteriorated measurement and suggested that nanoDots were limited to rotate under 30° for the measurement accuracy (under 4%).

The OSL dosimeter almost never showed energy dependence in the therapeutic energy range.^9^, ^16^ On the other hand, it has been shown that the OSL dosimeter over‐responded to low‐energy X‐rays.^7^, ^11^, ^12^ This can be attributed to the high photoelectric effect of Al_2_O_3_:C at low photon energy values, which raises its mass energy absorption coefficient relative to water.^11^ Al‐Senan and Hatab^11^ reported that the energy dependence in general radiography energy range was between 0.81 and 1.56. The range of energy dependence in this study was lower than that in general radiography energy range. Scarboro et al.^12^ reported that the variation in energy dependence for CT in air and a phantom was between + 20% and −15%. This energy dependence was higher than that of the result in this study. Hsu et al.^5^ reported a fitted curve and equation of energy dependence for thin film‐thermoluminescent dosimeters in the mammography energy range. The energy dependence of thin film‐thermoluminescent dosimeters in the range from 14.7–17.2 keV (Mo/Mo and Mo/Rh) calculated by the equation from Hsu et al.^5^ was converted to a CF between 0.95–1.01, based on Mo/Rh 28 kV conditions. It was almost the same as the CF range in this study (Mo/Mo 22–28 kV and Mo/Rh 28–32 kV, 0.94–1.01). Therefore, the energy dependences of the OSL dosimeter and thin film‐thermoluminescent dosimeter were similar. The CF range in Mo/Mo 26–28 kV and Mo/Rh 28–32 kV was 0.99–1.01, and the OSL dosimeter could be used for the same CF.

The angular dependence of the nanoDot was compared between three tube voltage and target/filter combinations. The greatest deterioration was about 60% when the X‐ray tube was rotated at a 90° angle compared with 0° for all directions. However, the deterioration of angle interval was not constant, the deterioration of over 60° increased more. The lower the effective energy, the higher was the deterioration of the signal counts in the angular dependence. More angled X‐rays were filtered out and attenuated before reaching the detector because an angled X‐ray travels a longer distance while passing through the plastic case than an X‐ray facing the detector's serial number. Al‐Senan and Hatab^11^ reported that the angular dependence of nanoDot dosimeters showed variations as high as 70% in mammography and were close to the result of this study, and angular dependence was the highest for other modalities, including therapeutic settings (under 5%),^17^ general radiography (under 40%),^11^ and CT (under 11%).^12^ In a breast tomosynthesis system, effective energy is higher than those of mammography and the X‐ray tube is rotated from 15° to 50°.^20^ NanoDots could accurately measure an X‐ray tube rotated under 60° in a tomosynthesis system (under 6%).

The commercial OSL dosimeter evaluated the energy dependence and the angular dependence based on the tube voltage of each target and filter combinations in the mammography energy range. Radiation dosimeter for quality control of mammography was required to calibrate at appropriate mammographic energies with an energy response within ± 5% and an accuracy within ± 5%, as per the IAEA Human Health Series No. 17.^21^ The energy response in the range from Mo/Mo 24 kV to W/Rh 32 kV in this study was within ± 5%. The energy dependence in the typical mammography energy range was not as much as that in general radiography or CT. The angular dependence under 30° in this study was within ± 5%. The OSL dosimeter calibrated at typical energy in the mammography energy range was usable to measure the entrance surface dose so as not to be tilted to allow for highly accurate measurement. In energy dependence and angular dependence, the commercial OSL dosimeter‐calibrated mammography energy range could be used for quality control of the radiation dosimeter.

## 5 Conclusions

The commercial OSL dosimeter evaluated the 2 specific dosimetric characteristics, which were energy and angular dependence, based on the tube voltage of each target and filter combinations in the mammography energy range. Energy dependence in the typical mammography energy range of the OSL dosimeter was lower than that in general radiography and CT, and was equal to that of thin film‐thermoluminescent dosimeters. Angular dependence showed the least measurement accuracy for all target and filter combinations. In energy dependence and angular dependence, the OSL dosimeter‐calibrated typical mammography energy was suggested to be used for accurate measurement at under 30° of the tilt.

## Conflict of interest

The authors declare that they have no conflict of interest.
